# Effects of icariin on the fracture healing in young and old rats and its mechanism

**DOI:** 10.1080/13880209.2021.1972121

**Published:** 2021-09-12

**Authors:** Xiaoyun Zhang, Yueping Chen, Chi Zhang, Xuan Zhang, Tian Xia, Jie Han, Shilei Song, Canhong Xu, Feng Chen

**Affiliations:** a Clinical Medical School, Jiangxi University of Traditional Chinese Medicine, Nanchang, China; b Department of Orthopedics, Ruikang Hospital Affiliated with Guangxi University of Chinese Medicine, Nanning, China

**Keywords:** *Epimedium*, rat bone mesenchymal stem cells, alkaline phosphatase, BMP-2/Smad5/Runx2 pathway

## Abstract

**Context:**

Icariin has attracted increasing attention because of its wide variety of pharmacological effects.

**Objective:**

This study investigates whether icariin could promote fracture healing in young and old rats and its mechanisms.

**Materials and methods:**

A Wistar rat model for the tibia fracture in relatively young and old rats, respectively, was established. The rats were divided into four groups: model group, L-icariin (50 mg/kg icariin), M-icariin (100 mg/kg icariin) and H-icariin (200 mg/kg icariin), and intragastric administration of icariin was performed for 10 days or 20 days. In addition, isolated and cultured rat bone mesenchymal stem cells (rBMSCs) from young and old rats were cultured with 5% and 20% of icariin-containing serum, respectively, then cell viability and alkaline phosphatase (ALP) activity were measured.

**Results:**

Icariin administration induced the expression of Runx2, Osterix, BMP-2, p-Smad5 and osteocalcin secretion (young rats: model: 2.50 ± 0.71; L-icariin: 10.10 ± 1.55; M-icariin: 24.95 ± 2.19; H-icariin: 36.80 ± 2.26; old rats: model: 1.55 ± 0.49; L-icariin:6.55 ± 0.50; M-icariin: 15.00 ± 0.85; H-icariin:20.50 ± 2.27) at the fracture site, and increased the levels of bone formation markers (OC, BAP, NTX-1 and CTX-1) in a dose-dependent manner. *In vitro*, icariin treatment promoted rBMSC viability, increased ALP activity and the expression of BMP-2/Smad5/Runx2 pathway proteins.

**Discussion and conclusions:**

Icariin may accelerate fracture healing by activating the BMP-2/Smad5/Runx2 pathway in relatively young and old rats. The research on the mechanism of icariin to promote fracture healing can provide a theoretical basis for the clinical application and promotion of icariin.

## Introduction

Currently, fractures are very common all over the world. Healing in the vast majority of patients with fractures occurs by natural healing rather than by accelerated healing, and natural healing involves a long and slow physiological process (Giannoudis, Tzioupis, et al. 2007; Dimitriou et al. 2011). Therefore, it is necessary to find and study drugs or interventions that can accelerate fracture healing, and recent studies have identified certain substances that can indeed accelerate this process (Giannoudis, Psarakis, et al. 2007). Xu et al. (2015) pointed out that Sry-related high-mobility group box 11 can accelerate fracture healing and reduce delayed unions or nonunions by regulating mesenchymal stem cell differentiation and migration. Additionally, traditional Chinese medicine has been widely used in the treatment of fractures, osteoporosis and other bone-related diseases for thousands of years, and they can stimulate bone regeneration and accelerate fracture healing (Zhou et al. 2004). The roles of traditional Chinese medicine, such as *Epimedium*, in treating fractures have attracted researchers’ attention (Huang and You 1997; Zhang, Niu, et al. 2017).

*Epimedium brevicornu* Maxim. (Berberidaceae) (*Epimedium*) has been used in Chinese ethnopharmacology for the treatment of metabolic bone diseases. Icariin is a flavonoid and the active compound in *Epimedium*, which has been used to cure different diseases, including bone fractures, osteoporosis and tumours (Iqbal et al. 2018; Wang et al. 2018). In osteoporosis and osteoporotic fractures with different causes, oral medication of icariin can promote bone formation by inhibiting bone resorption and improve peak bone mineral density and bone quality (Cheng et al. 2014). Wu et al. (2018) found that icariin promoted bone formation, inhibited bone loss and effectively restored bone structure and strength in chronic high-dose alcohol-induced osteopenic rats. *In vitro*, icariin promoted the proliferation of rat BMSCs by activating the ERK and p38 MAPK signalling pathways (Qin et al. 2015). Despite the increase in the clinical usage of the *Epimedium* plant genus, the mechanisms underlying its therapeutic effects of the pharmacologically active constituent (icariin) on fracture healing are still not clearly understood.

The BMP-2/Smad5/Runx2 pathway is one of the most important pathways in osteoblast proliferation and differentiation (Dai et al. 2013). Based on this information, we suspect that the therapeutic effects of icariin on fracture healing are related to the BMP-2/Smad5/Runx2 pathway. Therefore, we established a rat model of fracture in relatively young and old rats, and isolated rat bone mesenchymal stem cells (rBMSCs) from the model rats and then intragastrically administered icariin or cocultured the cells with it. We detected indicators that indicate fracture healing to ultimately reveal the underlying mechanisms of the therapeutic effects of icariin on fracture healing in relatively young and old rats.

## Materials and methods

### Animals and regents

Twenty-four 6-month-old Wistar rats (male/female 1:1; 280–330 g) and twenty-four 22-month-old Wistar rats (male/female 1:1; 440–500 g) were purchased from the Medical Laboratory Animal Center of Guangdong Province. The experimental studies were approved by the Institutional Animal Care and Use Committee of Guangxi University of Chinese Medicine (approval no. DW20180328-31). The rats were housed in a temperature-controlled environment (25 °C ± 2 °C) with a 12 h light/dark cycle and free access to food and water. The icariin standard (HPLC > 94%) was purchased from Sigma-Aldrich (#I1286, Shanghai, China).

### Modelling of fractures caused by trauma in young and old rats, and treatment

Forty-eight Wistar rats (6 months and 22 months) were anaesthetized using 2% isoflurane, respectively. After animals were successfully anaesthetized, 1–3 pounds of weight were dropped from a height (10–15 cm) on the middle of the tibia, causing a tibia fracture. Postoperative intraperitoneal injection of painkillers and antibiotics were provided to prevent postoperative pain and infection after modelling.

After generating the fracture model, the rats were randomly divided into four groups: model group (gavaged 1 mL saline), low-dose group (L-icariin, gavaged 50 mg/kg icariin in 1 mL saline), medium-dose group (M-icariin, gavaged 100 mg/kg icariin in 1 mL saline) and high-dose group (H-icariin, gavaged 200 mg/kg icariin in 1 mL saline), with six rats in each group. The above intragastric administration was prepared once a day for a total of 10 days or 20 days. All rats were free to drink water. The doses of icariin (50, 100 and 200 mg/kg) were obtained from clinical experience and preliminary experiment in rats.

### Sampling methods

On the 1st day, 10th day and 20th day, the rats were subjected to digital radiography (DR) examination (Goodsee, #GDA32-02, China) of their fracture healing, and scored the degree of fracture healing using the Lane–Sandhu scoring standard (Wang et al. 2007). On the 10th day, three rats of each group were anaesthetized using 2% isoflurane; the coeliac arterial blood was immediately isolated and centrifuged at 5000 rpm for 10 min, and the serum was used for ELISA assay. Then, the rats were euthanized by exsanguination under 2% isoflurane anaesthesia. The entire right tibia was taken for histological examination, RT-PCR and Western blot.

### Haematoxylin and eosin (HE) staining

The entire right tibia was removed and fixed in 4% paraformaldehyde-phosphate-buffered saline (PBS) solution for three days. After decalcification of the entire right tibia, it was embedded in paraffin and then cut into 5 μm sections. The sections were stained as follows: 70% ethyl alcohol for 10 s, diethylpyrocarbonate-treated water for 5 s, haematoxylin with RNase inhibitor for 20 s, 70% ethyl alcohol for 30 s and eosin Y in 100% ethyl alcohol for 20 s, followed by dehydration in a series of alcohol for 30 s each and in xylenes for 2 min. HE staining images were captured at ×400 magnification (CX71, Olympus Corporation, Tokyo, Japan).

### RNA preparation and RT-PCR

The bone tissue was located at the fracture site, placed in liquid nitrogen, and ground; then, the total RNA was extracted by using TRIzol reagent (Invitrogen, Carlsbad, CA). cDNA was synthesized using a cDNA Synthesis Kit (Takara, Dalian, China). Quantitative RT-PCR (qRT-PCR) was performed to detect the expression levels of mRNA using SYBR Green and a LightCycler 480 detection system (Roche Diagnostics, Indianapolis, IN). GAPDH mRNA levels were used for normalization. The primer sequences were as follows: GAPDH: 5′-TGACAACTTTGGCATCGTGG-3A and 5′-GGGCCATCCACAGTCTTCTG-3GC Runx2: 5′-GCCTTCAAGGTTGTAGCCCT-3C and 5′-TGAACCTGGCCACTTGGTTT-3AA BMP-2: 5′-GGACGTCCTCAGCGAGTTT-3A and 5′-CAGGTCGAGCATATAGGGGG-3GGTOsterix: 5′-GGCTGAGGAAGAAGCCCATT-3C and 5′-AAGTGGGCTTTCAGATGCGA-3GT Smad5: 5′-TGTTGGGCTGGAAACAAGGT-3T and 5′-GTGACACACTTGCTTGGCTG-3GAC. qRT-PCR results were analysed and expressed as relative mRNA levels based on the CT value, which was then converted to fold change.

### Western blot assay

The bone tissue was located at the fracture site, placed in liquid nitrogen, and ground; then, it was lysed using lysis buffer, and the total protein concentration of lysates was measured using a micro BCA protein assay kit (Pierce, Rockford, IL). The samples were separated on 12% SDS-polyacrylamide gels and then electrophoretically transferred to polyvinylidene difluoride membranes. After blocking in 5% non-fat milk solution for 1 h, the membranes were incubated with anti-Osterix (Abcam, ab209484, Cambridge, MA, dilution 1:1000), anti-BMP-2 (Abcam, ab14933, Cambridge, MA, dilution 1:500), anti-phosphorylated-Smad5 (Abcam, ab92698, Cambridge, MA, dilution 1:200), anti-Smad5 (Abcam, ab40771, Cambridge, MA, dilution 1:1000), anti-Runx2 (Abcam, ab76956, Cambridge, MA, dilution 1:200) and GAPDH antibodies for 2 h at 37 °C. Then, the membranes were washed three times and incubated with horseradish peroxidase-conjugated secondary antibodies for 1 h at room temperature. Next, the membranes were treated with ECL solution (Millipore, Darmstadt, Germany) and then imaged using ImageJ (Bethesda, MD). The grey values of each protein band were measured by Photoshop CS5 software (Adobe, San Jose, CA). The relative band intensity was assessed as the ratio of the grey value of each protein to that of the corresponding GAPDH.

### Immunohistochemistry assay

After decalcifying the entire right tibia, it was embedded in paraffin and then cut into 5 μm sections. Following hydration and blocking, the slides were treated with peroxide. An anti-osteocalcin antibody (Abcam, ab13420, Cambridge, MA, dilution 1:500) was incubated with the slides at 37 °C for 2 h. After incubating with a secondary antibody at 37 °C for 1 h, the slides were treated with 3,3′-diaminobenzidine (DAB) solution. Finally, the slides were lightly counterstained with haematoxylin and dehydrated. Visual analysis was performed with an Olympus fluorescence microscope (Olympus, CX71, Tokyo, Japan) at ×400 magnification. The medium optical density (MOD) of osteocalcin expression in each group was analysed by Image-Pro Plus software (Media Cybernetics, Rockville, MD).

### ELISA

A Rat OC ELISA kit (Cloud-Clone Corp., #SEA471Ra, Wuhan, China), Rat BAP ELISA kit (Jiancheng, #A059-1, Nanjing, China), Rat NTX-1 ELISA kit (Cloud-Clone Corp., #CEA639Ra, Wuhan, China) and Rat CTX-1 ELISA kit (Cloud-Clone Corp., Wuhan, #CEA665Ra, China) were used for OC, BAP, NTX-1 and CTX-1 measurements in this study. All assays were performed exactly following the manufacturer’s instructions.

### Isolation, culture and identification of rBMSCs

Six-month-old/22-month-old rats were sacrificed by euthanized by exsanguination under 2% isoflurane anaesthesia. Under sterile conditions, 1 mL of bone marrow was extracted from the femur and tibia using a syringe prefilled with 2 mL of heparin for anticoagulation. Bone marrow mononuclear cells were separated by gradient density centrifugation and cultured in osteogenic differentiation medium of Wistar rat BMSCs (Cyagen Biosciences, RAWMX-90021, Santa Clara, CA) supplemented with 20% foetal bovine serum (HyClone, Logan, UT), 100 U/mL penicillin and 100 U/mL streptomycin at 37 °C in a 5% CO_2_ atmosphere (Thermo, Waltham, MA). Forty-eight hours later, nonadherent cells were removed, and the adherent cells were cultured and expanded in another flask. After 2–3 weeks of continuous culture, homogenous rBMSCs from young and old animals were obtained and identified by immunofluorescence.

The identification of rBMSCs was performed by immunofluorescence using a fluorescently labelled antibody. rBMSCs were fixed in 4% (w/v) paraformaldehyde in PBS for 20 min and then incubated with anti-CD29-FITC (eBioscience, #11-0291-82, Shanghai, China), anti-CD44-FITC (eBioscience, #MA5-17522, Shanghai, China) and anti-CD45-FITC (eBioscience, #11-0461-82, Shanghai, China). Visual analysis was performed with an Olympus fluorescence microscope (Olympus, CX71, Tokyo, Japan) at ×400 magnification.

### Preparation of icariin-containing serum

Six adult Wistar rats (male/female 1:1; weight, 280–330 g, 6 months old) were orally gavaged with 200 mg/kg icariin once a day for seven days (Zhang et al. 2020). After seven days, the rats were euthanized by exsanguination under 2% isoflurane anaesthesia, and blood samples were collected. The icariin-containing serum was prepared by centrifugation at 2000×*g* at 4 °C for 15 min. rBMSCs were cultured with 5% and 20% icariin-containing serum or 5% and 20% icariin-free serum, and then RT-PCR, Western blot, CCK-8 and alkaline phosphatase (ALP) activity assays were performed.

### CCK-8 assay

Homogenous rBMSCs from young and old animals were seeded in triplicate in 96-well plates (5 × 10^3^ cells/well), and cultured in osteogenic differentiation medium supplemented with 5% and 20% icariin-containing serum, or 5% and 20% icariin-free serum for 72, 96 and 120 h, respectively. After treatment, the CCK-8 working solution was added to the wells for 1 h of incubation, and then cell viability was assessed daily by absorbance at 490 nm using a microplate reader (model 680 Microplate Reader, Bio-Rad, Hercules, CA).

### ALP activity assay

rBMSCs from young and old animals were seeded in triplicate in 24-well plates (5 × 10^4^ cells/well), treated with 5% and 20% icariin-containing serum and 5% and 20% icariin-free serum for 120 h, and then the ALP activity of the different rBMSC was detected. ALP activity was determined using an ALP activity kit (Jiancheng, #A059-1, Nanjing, China).

### Statistical methods

Data were statistically analysed using SPSS 17.0 (SPSS Inc., Chicago, IL). All results are presented as the mean values ± standard deviations. *p< 0.05, **p< 0.01 and ***p< 0.001 were considered statistically significant. Statistically significant differences between groups were determined by one-way analysis of variance (ANOVA) with the Bonferroni *post hoc* test when equal variances were assumed, or with Dunnett's T3 *post hoc* test when equal variances were not assumed. Figures were graphed using GraphPad Prism 5 (GraphPad Software, La Jolla, CA).

## Results

### Icariin accelerated fracture healing

**Figure 1.**
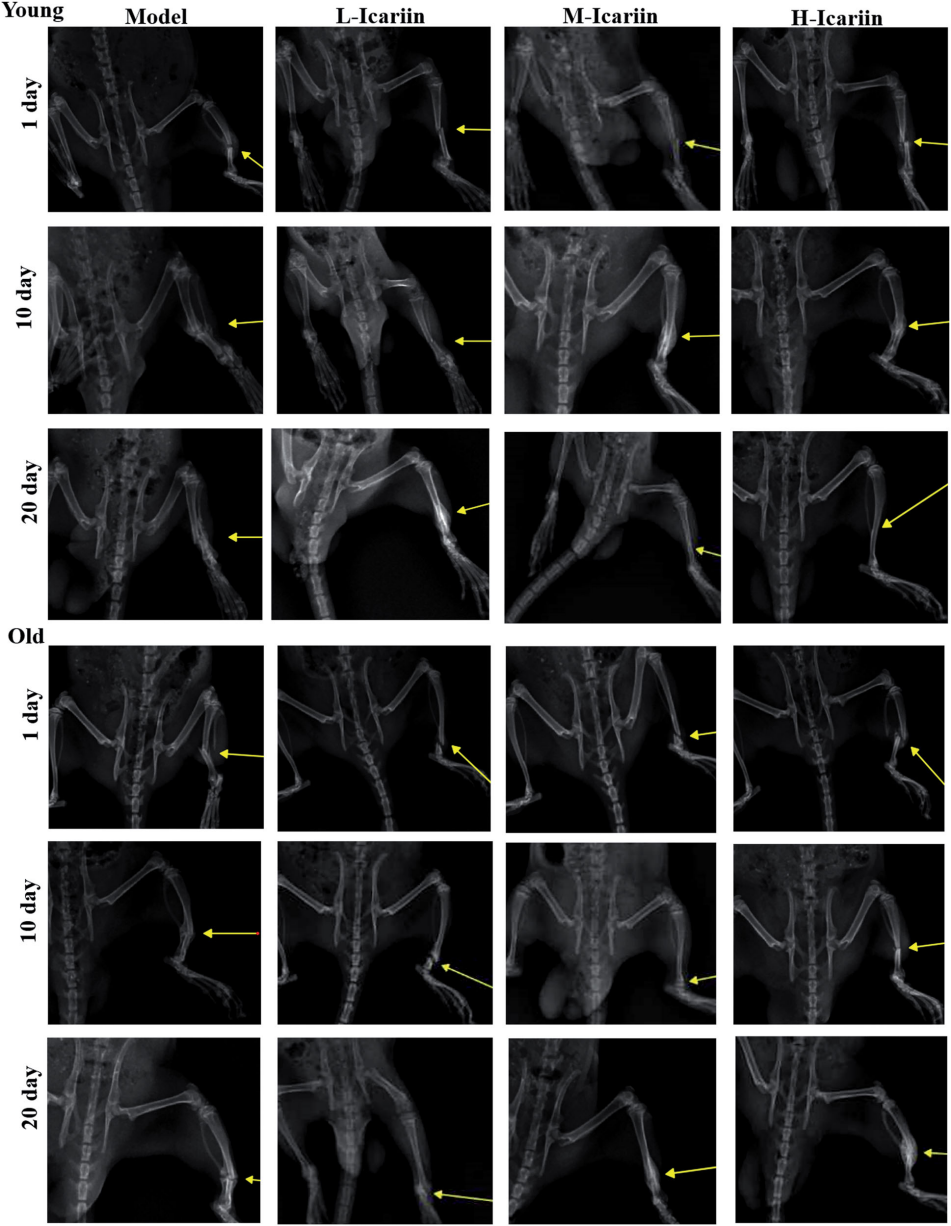
Icariin accelerated fracture healing in the rat model of fractures. Digital radiography (DR) was used to observe fracture healing on the 1st day, 10th day and 20th day.

DR of rat tibias showed incomplete healing in rats subjected to natural healing on the 1st day, 10th day and 20th day in both young and old rats. Compared with the model rats with natural healing, at the 10th day and 20th day, the fracture model rats treated with icariin exhibited better fracture healing and Lane–Sandhu’s scores (Table 1). Additionally, the fracture healing speed and Lane–Sandhu’s scores of young rats were higher and faster than that of old rats (Table 1). DR images (Figure 1) indicated that icariin can accelerate fracture healing in both young and old rat models of fractures in a dose-dependent manner.

**Table 1.** Lane–Sandhu’s scores of DR in each group.

Groups	Time (days)	*n*	Model	L-Icariin	M-Icariin	H-Icariin
Young	1	6	0.00 ± 0.00	0.00 ± 0.00	0.00 ± 0.00	0.00 ± 0.00
	10	6	2.33 ± 0.52	3.33 ± 0.52^#^	5.16 ± 0.41*^,#^	5.50 ± 0.55*^,#^
	20	3	4.67 ± 0.58	5.33 ± 0.58^#^	8.33 ± 0.58*^,#^	9.33 ± 0.58*^,#^
Old	1	6	0.00 ± 0.00	0.00 ± 0.00	0.00 ± 0.00	0.00 ± 0.00
	10	6	2.17 ± 0.41	2.33 ± 0.52	4.17 ± 0.41*	4.83 ± 0.41*
	20	3	4.00 ± 1.00	4.33 ± 0.58	5.00 ± 0.00*	5.67 ± 0.58*

* *p*< 0.05 vs. corresponding time’s model group.

# *p*< 0.05 vs. corresponding time’s old group.

**Figure 2.**
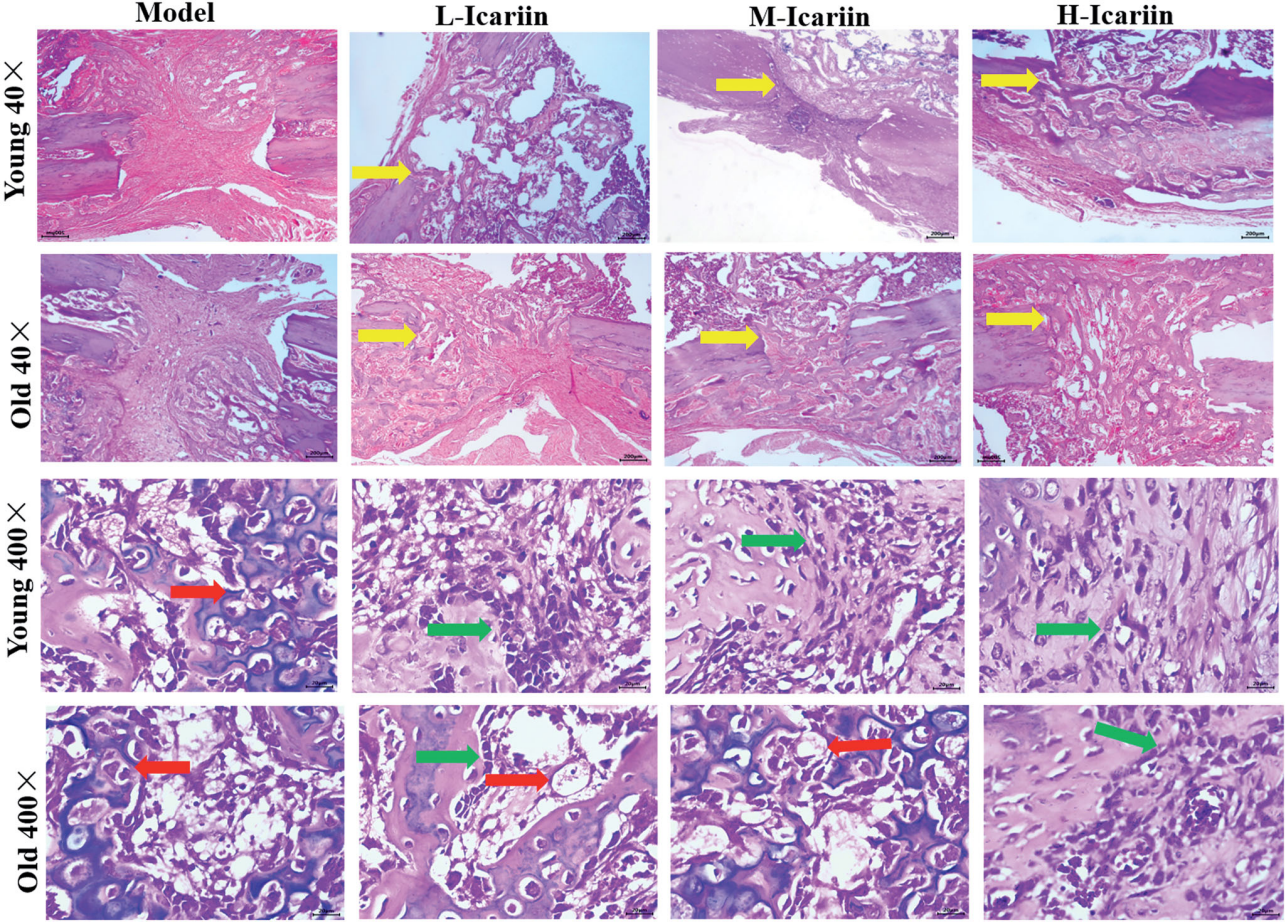
HE staining images of the fracture site in the rat model of fractures. Magnification ×40 and ×400. Yellow arrow: trabecular bone. Red arrow: chondrocytes. Green arrow: osteoblasts.

After 10 days of icariin administration, compared with the model group with natural healing and the L-icariin group, low magnification (×40) images showed faster transformation of trabecular bone into compact bone in the H-icariin group, and high magnification (×400) images showed that the H-icariin group had more osteoblasts and faster compact bone substance formation. Additionally, compared with the same treatment, the number of osteoblasts and bone marrow cells in young rats was higher than it was in old rats. DR (Figure 1) and HE staining (Figure 2) indicated that icariin can induce bone formation and accelerate fracture healing in young and old rat models of fracture in a dose-dependent manner.

### Icariin activated the BMP-2/Smad5/Runx2 pathway in the rat fracture model

**Figure 3.**
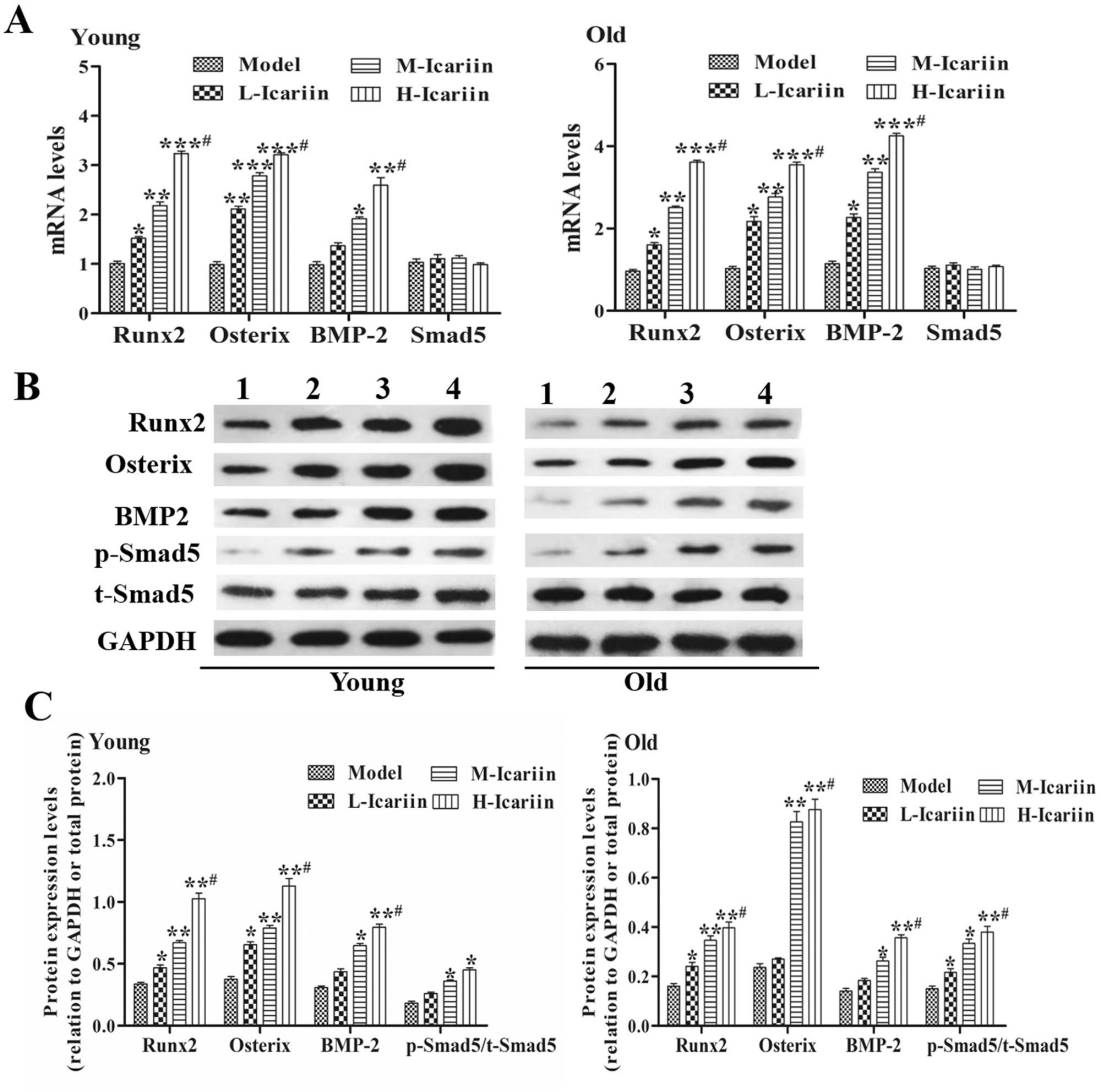
Icariin activates the BMP-2/Smad5/Runx2 pathway in the rat model of fractures. (A) RT-qPCR was performed to detect the mRNA expression levels of Runx2, Osterix, BMP-2 and Smad5. GAPDH was used as the control. (B) The expression levels of Runx2, Osterix, BMP-2, p-Smad5 and t-Smad5 proteins were detected by Western blotting. (1) Model group, (2) L-icariin group, (3) M-icariin group and (4) H-icariin group. (C) The relative expression levels of Runx2, Osterix, BMP-2, p-Smad5 and t-Smad5 were calculated by normalizing to the levels of GAPDH. Data are represented as the means ± SD (*n* = 3). *p< 0.05, **p< 0.01 and ***p< 0.001 vs. the model group. *^#^p*< 0.05 vs. the corresponding L-icariin group.

RT-PCR and Western blot detected the expression levels of Runx2, Osterix, BMP-2 and Smad5 mRNAs and proteins in rat fracture models (Figure 3). In the young rat fracture models, after 10 days of icariin administration, the levels of Runx2, Osterix, BMP-2 and p-Smad5 in the fracture section were significantly higher than those of the model group (*p* < 0.05). Compared with the L-Icariin group, the levels of Runx2, Osterix, BMP-2 and p-Smad5 in the H-Icariin group were significantly increased. Additionally, icariin presented the same effects in the old rat fracture models. These data showed that icariin can activate the BMP-2/Smad5/Runx2 pathway in a dose-dependent manner.

### Icariin regulated the levels of bone formation- and bone resorption-specific markers in rat fracture models

**Figure 4.**
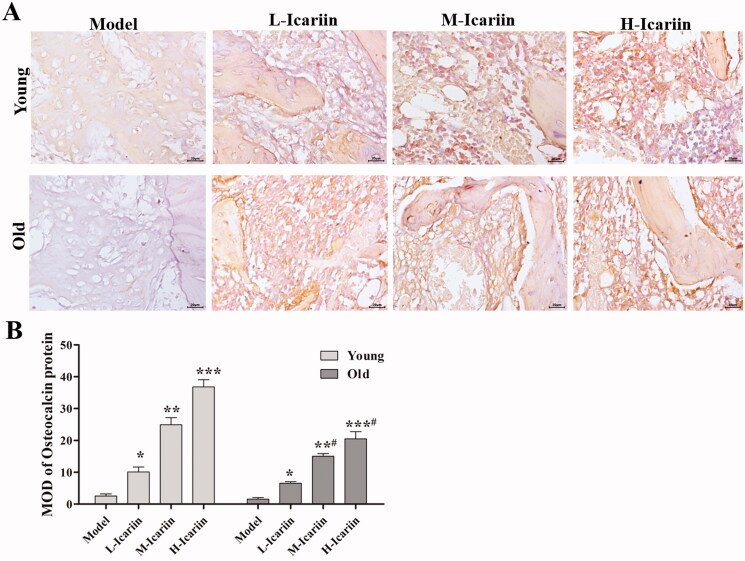
Icariin induces the expression of osteocalcin in the rat model of fractures. (A) Immunohistochemistry to detect the expression of osteocalcin protein. (B) The MOD of osteocalcin expression was calculated using Image-Pro Plus 6.0 software. Data are represented as the means ± SD (*n* = 3). *p< 0.05, **p< 0.01 and ***p< 0.001 vs. the model group. *^#^p*< 0.05 vs. the corresponding young group.

We detected the levels of bone formation- and bone resorption-specific markers. The results in Table 2 show that the levels of OC, BAP, NTX-1 and NCTX-1 in icariin-treated rats were critically higher than they were in model rats. Surprisingly, compared with the L-Icariin group, the levels of OC, BAP, NTX-1 and NCTX-1 in the H-Icariin group were significantly increased. Similar results were also obtained both rat fracture models. In addition, we measured the expression levels of OC at the fracture section using an immunohistochemistry assay on the 10th day and found that icariin treatment induced OC expression in a dose-dependent manner (Figure 4). These results revealed that icariin could accelerate bone formation and bone resorption in the young and old fracture rats.

**Table 2.** The concentrations of OC, BAP, NTX-1 and CTX-1 in our rat model of fractures on the 10th day (*n* = 3).

Groups	Markers	Model	L-Icariin	M-Icariin	H-Icariin
Young	OC (ng/mL)	1.78 ± 0.065	1.79 ± 0.067	1.83 ± 0.057	2.27 ± 0.064*^,#^
	BAP (U/mg prot)	36.3 ± 1.57	41.05 ± 1.81	48.86 ± 1.10*	60.85 ± 2.03*^,#^
	NTX-1 (ng/mL)	1.05 ± 0.057	1.15 ± 0.062	1.21 ± 0.062*	1.59 ± 0.071*^,#^
	CTX-1 (ng/mL)	0.50 ± 0.041	0.55 ± 0.048	0.67 ± 0.052*	1.18 ± 0.067*^,#^
Old	OC (ng/mL)	0.94 ± 0.058	1.42 ± 0.061*	1.51 ± 0.063*	1.86 ± 0.064*^,#^
	BAP (U/mg prot)	22.45 ± 1.64	25.34 ± 2.15	32.69 ± 1.50*	49.65 ± 2.41*^,#^
	NTX-1 (ng/mL)	0.71 ± 0.052	0.85 ± 0.049	0.95 ± 0.058*	1.20 ± 0.063*^,#^
	CTX-1 (ng/mL)	0.28 ± 0.058	0.41 ± 0.053*	0.51 ± 0.055*	0.96 ± 0.053*^,#^

Data are represented as the means ± SD (*n* = 3).

* *p*< 0.05 vs. model group.

# *p*< 0.05 vs. L-Icariin group.

### Icariin increased cell viability and ALP activity in rBMSCs from young and old rats

**Figure 5.**
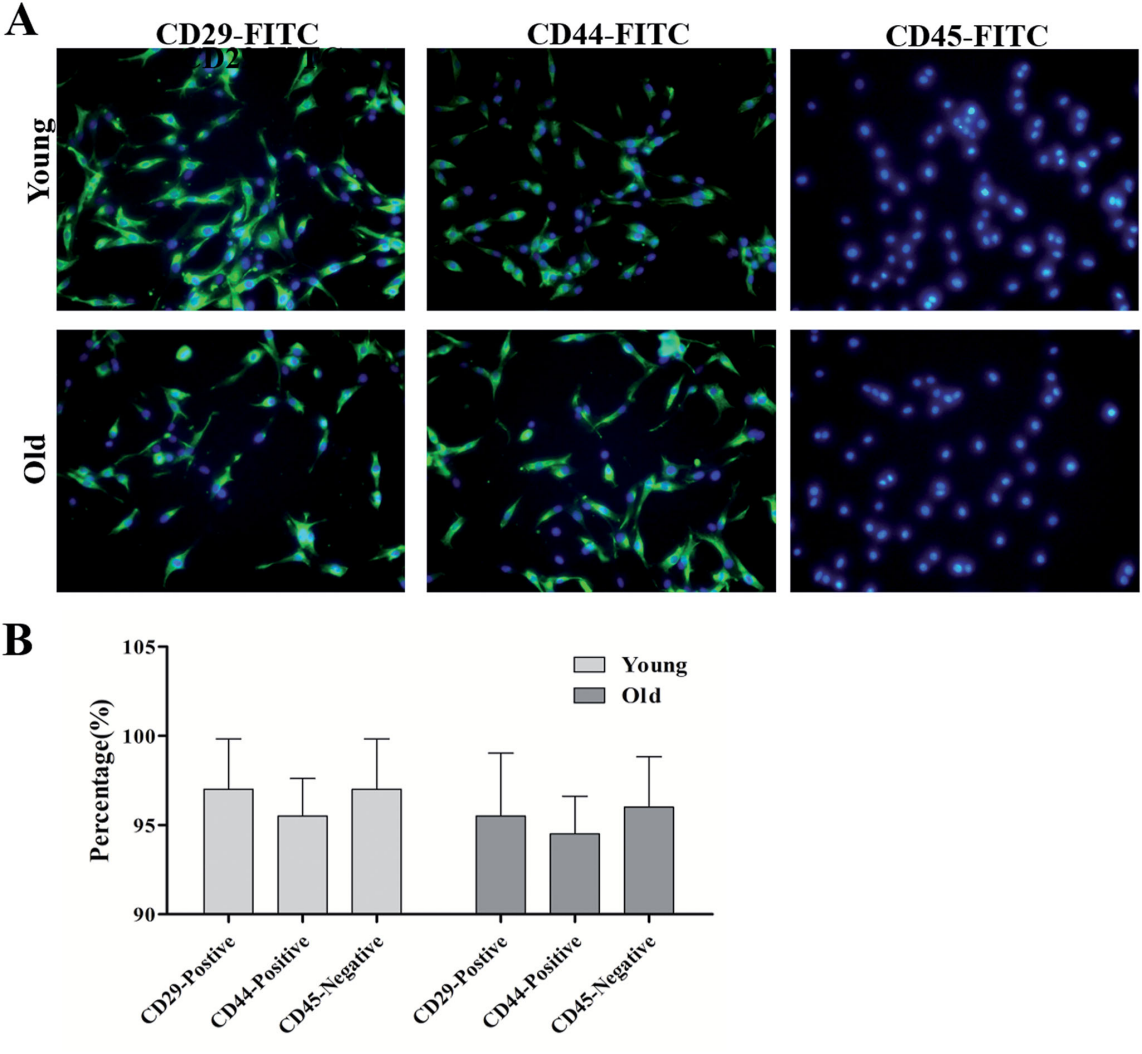
Immunofluorescence identification of the rBMSCs isolated from young and old rats. (A) Immunofluorescence was used to identify the purity of rBMSCs using a fluorescently labelled antibody. (B) The percentage of CD29-positive, CD44-positive and CD45-negative rBMSCs from young and old rats. Magnification ×400. Data are represented as the means ± SD (*n* = 5).

After isolation of the rBMSCs from young and old rats, immunofluorescence was used to identify the purity of the rBMSCs using a fluorescently labelled antibody. As shown in Figure 5(A), the percentage of CD29-positive and CD44-positive rBMSCs exceeded 95%, and the percentage of CD45-negative rBMSCs exceeded 95% (Figure 5(B)). Similar results were also obtained in rBMSCs from both young and old rats. Immuophenotypic studies have shown that cultured rBMSCs are CD29- and CD44-positive and CD45-negative (Qin et al. 2015; Wu et al. 2018). Therefore, these data show that the purity of rBMSCs (95%) was high enough and could be used for follow-up experiments.

**Figure 6.**
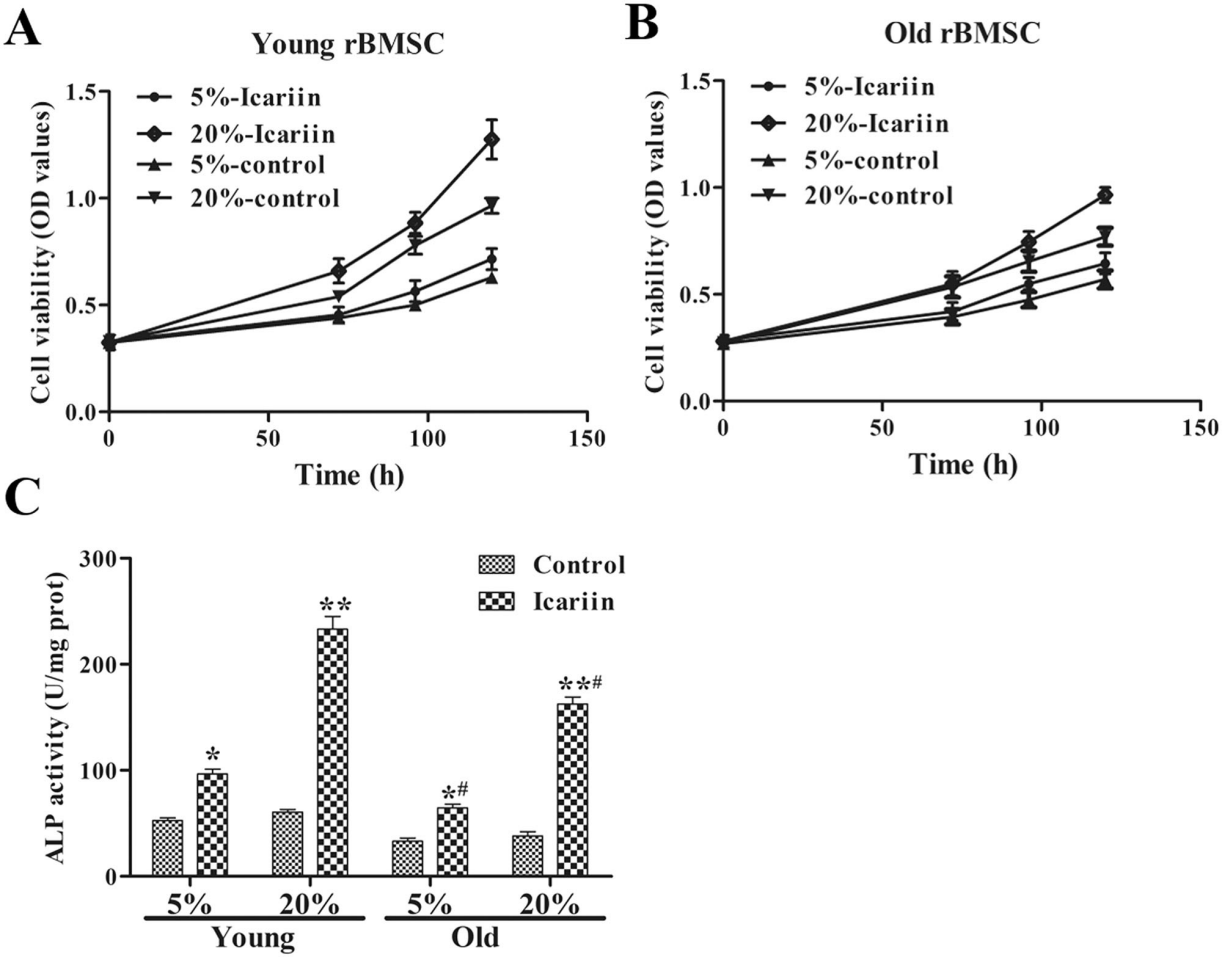
The rBMSC viability (A, B) and ALP activity (C) assay of rBMSCs from young and old rats after icariin treatment. Data are represented as the means ± SD (*n* = 6). *p< 0.05; **p< 0.01 and *^#^p*< 0.05 vs. its control group.

Then, we performed CCK-8 and ALP activity assays to show the effects of icariin on rBMSC viability and ALP activity. As shown in Figure 5, at the same serum concentration (5% and 20%), the rBMSC viability and ALP activity of the icariin-containing serum group were significantly higher than those of the icariin-free serum group. For the various concentrations of icariin-containing serum (5% and 20%), rBMSC viability and ALP activity were upregulated (Figure 6). Surprisingly, compared with young rBMSCs, rBMSC viability and ALP activity in older rBMSCs were significantly decreased. These results revealed that icariin increased rBMSC viability and ALP activity in rBMSCs from young and old rats.

### Icariin activated the BMP-2/Smad5/Runx2 pathway in rBMSCs from young and old rats

**Figure 7.**
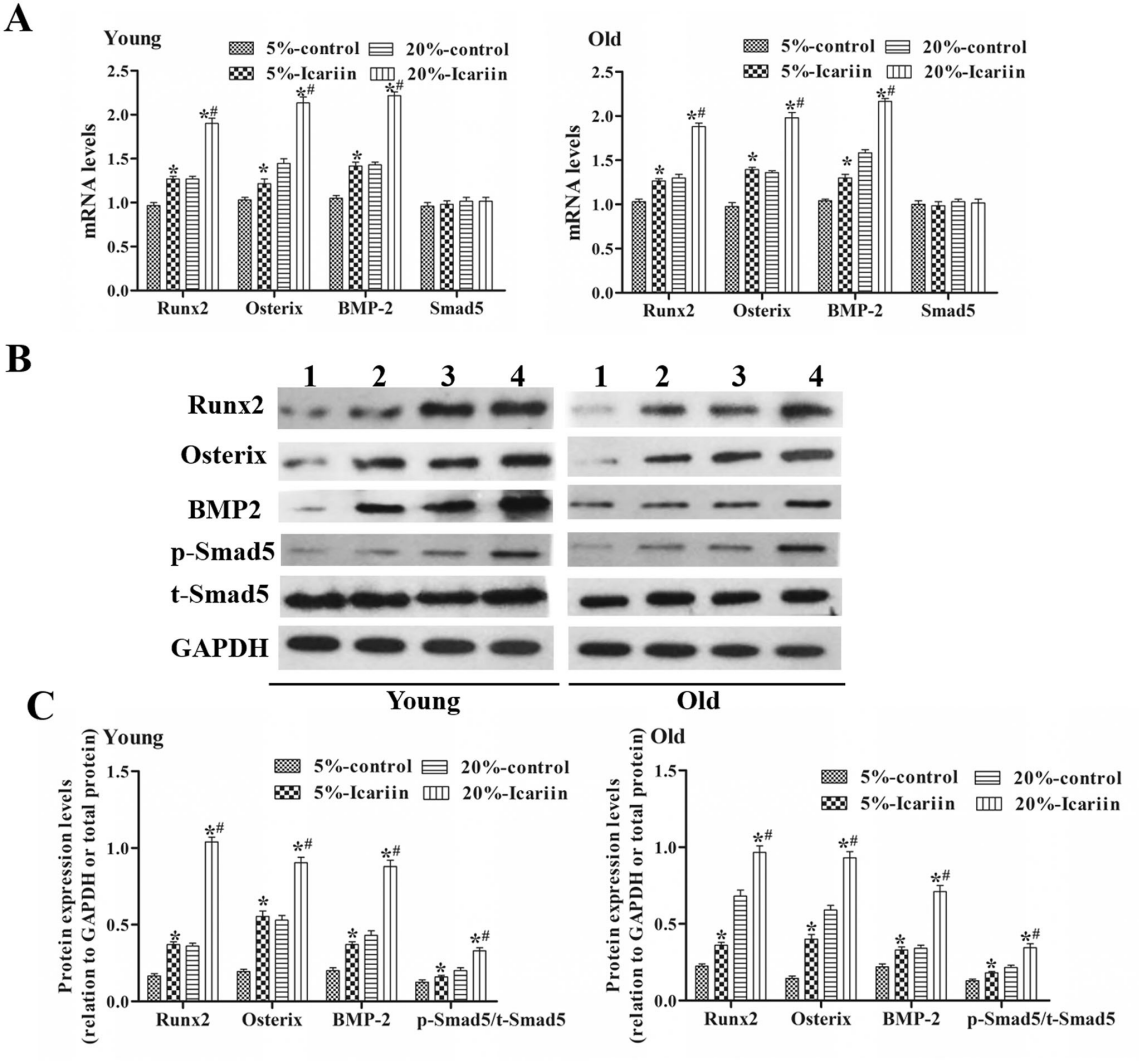
Icariin activated the BMP-2/Smad5/Runx2 pathway in rBMSCs from young and old rats. (A) RT-qPCR was performed to detect the mRNA expression levels of Runx2, Osterix, BMP-2 and Smad5. GAPDH was used as the control. (B) The expression levels of Runx2, Osterix, BMP-2, p-Smad5 and t-Smad5 proteins were detected by Western blotting. (1) 5% icariin-control group, (2) 5% icariin-containing group, (3) 20% icariin-control group and (4) 20% icariin-containing group. (C) The relative expression levels of Runx2, Osterix, BMP-2, p-Smad5 and t-Smad5 were calculated by normalizing to the levels of GAPDH. Data are represented as the means ± SD (*n* = 4). *p< 0.05 vs. its icariin-control group. *^#^p*< 0.05 vs. the 5% icariin-containing group.

RT-PCR and Western blot experiments were used to detect the expression levels of Runx2, Osterix, BMP-2 and Smad5 mRNAs and proteins in rBMSCs from young and old rats (Figure 7). Compared with the rBMSCs cultured in icariin-free serum, the levels of Runx2, Osterix and BMP-2 in rBMSCs cultured with 5% and 20% icariin-containing serum were significantly higher (*p* < 0.05). Compared with the 5% icariin-containing serum group, the levels of Runx2, Osterix and BMP-2 in the 20% icariin-containing serum group were significantly increased. Additionally, icariin exhibited the same effects on rBMSCs from young and old rats, and icariin promoted Smad5 phosphorylation. These data showed that icariin can activate the BMP-2/Smad5/Runx2 pathway in rBMSCs from young and old rats.

## Discussion

*Epimedium* has been used as a medicine in China for thousands of years, and its pharmacological effects and underlying mechanism are currently being studied in depth (Dai et al. 2013; Ti et al. 2017; Xi et al. 2018). Various studies have shown that icariin, as the major active component in *Epimedium*, has important pharmacological effects on metabolic bone diseases, including bone fractures and osteoporosis (Cheng et al. 2014; Qin et al. 2015; Wang et al. 2018; Wu et al. 2018). Our team studied the effects of icariin on fracture healing in relatively young and old rats, respectively, *in vitro* and *in vivo*. After intragastric administration of icariin for 10 days, we found that icariin could increase the levels of OC, BAP, NTX-1 and CTX-1 in serum, induce the expression of Runx2, Osterix, BMP-2 and p-Smad5, and promote viability and increase ALP activity in rBMSCs from young and old rats. In summary, our study showed that the therapeutic mechanism of icariin on fracture healing in relatively young and old rats.

Under normal circumstances, bone formation markers (e.g., OC and BAP) and bone resorption markers (e.g., NTX-1, CTX-1 and TRACP-5) are at relatively low levels in blood. When fractures occur, their levels increase significantly, and when the fractures heal, their levels return to normal levels (Froberg et al. 1999). Among them, OC, a secreted osteoblast-specific protein, is a unique noncollagenous protein of the extracellular matrix of bone that circulates in blood, and serum markers such as OC and ALP reflect osteoblastic activity in part during bone formation (Taniguchi et al. 2003; Bailey et al. 2017). Osteocalcin levels increased both in the bone and blood in femoral fracture model rats, and the higher the levels were, the faster the fracture healed (Aydin et al. 2014). In this study, we found that icariin could upregulate the levels of OC, BAP NTX-1 and CTX-1 in serum and increase OC expression in bone. These results indicate that icariin could regulate bone formation and bone resorption, resulting in the regulation of fracture healing.

The BMP-2/Smad5/Runx2 pathway is one of the most important pathways in bone metabolism. Bone morphogenetic proteins (BMPs) trigger intracellular signalling and activate Smad complexes that regulate the transcription of BMP-responsive genes, including Runx2 (Leboy et al. 2001). Runx2 (Cbfαl) is a transcription factor required for bone formation, and it functions synergistically with Smad1 and Smad5 to regulate bone-specific genes when BMPs induce osteogenesis (Ito and Miyazono 2003). A previous study indicated that the BMP-2/RUNX2 pathway is involved in osteogenic differentiation and mineralization of MC3T3-E1 osteoblastic cells *in vitro* and that it protects against ovariectomy-induced bone loss *in vivo* (Li et al. 2016). In ovariectomized rats, *Epimedium* promoted cartilage ossification of the callus and enhanced bone strength and bone quality via the CKIP-1/Runx2 pathway in the process of fracture healing (Shi et al. 2017). In bone infection caused by *Staphylococcus aureus*, Zhang, Shen, et al. (2017) found that icariin promoted bone formation by upregulating the BMP2/Runx2 and OPG/RANKL pathways under high local concentrations of vancomycin treatment. This study found that icariin can induce the expression of Runx2, Osterix, BMP-2 and p-Smad5 in our rat fracture model in a dose-dependent manner. Based on the roles of the BMP2/Runx2 pathway in bone metabolism, our results implied that icariin regulates bone formation and fracture healing via the BMP-2/Smad5/Runx2 pathway.

With the exception of haematopoietic stem cells, bone mesenchymal stem cells (BMSCs) are the main cells in the bone matrix environment, and they can differentiate into multiple types of cells, such as those in the chondrocytic and osteocytic lineages (Deans and Moseley 2002). It is becoming increasingly well accepted that BMSCs are involved in fracture repair and the bone regeneration process. When a fracture occurs, it is accompanied by the systemic mobilization of osteoblastic precursors to the fracture site, and increasing the number of BMSCs can accelerate fracture healing (Huang et al. 2015). ALP is mainly a biochemical marker of bone turnover, and it is usually used to monitor metabolic bone disease; higher serum ALP levels are associated with the incidence of fracture (Maruyama et al. 2014). A previous study pointed out that icariin enhanced human BMSC proliferation, motility and osteogenic differentiation through the STAT-3/CXCR4 pathway, and icariin may be a potential therapeutic for improving bone health (Lim et al. 2018). Our study found that icariin coculture could promote rBMSC viability, increase ALP activity and activate the BMP-2/Smad5/Runx2 pathway in rBMSCs from young and old animals, which indicates that icariin may induce osteogenic proliferation and differentiation of BMSCs by increasing ALP activity and activating the BMP-2/Smad5/Runx2 pathway.

## Conclusions

This study found that icariin may accelerate fracture healing in relatively young and old rats, respectively, by activating the BMP-2/Smad5/Runx2 pathway. Therefore, *Epimedium* and its active component icariin can be developed as a fracture healing treatment drug. In the future, our team will study the regulatory relationships between icariin and the BMP-2/Smad5/Runx2 pathway and will explore in depth other relevant molecular mechanisms.
